# Helminth diversity, prevalence, and host-specific patterns in wild and domestic ruminants of the Bukhara region, Uzbekistan

**DOI:** 10.5455/javar.2026.m1031

**Published:** 2026-03-24

**Authors:** Firuza D. Akramova, Adolat U. Mirzaeva, Shoira O. Saidova, Farangiz S. Uralova, Polot Y. Toshev, Ulugbek A. Shakarbaev, Djalaliddin A. Azimov, Mourad Ben Said, Hanène Belkahia

**Affiliations:** 1 Institute of Zoology, Academy of Sciences of the Republic of Uzbekistan, Tashkent, Uzbekistan; 2 Bukhara Specialized “Jeyran” Nursery, Bukhara, Uzbekistan; 3 Alfraganus University, Tashkent, Uzbekistan; 4 Laboratory of Microbiology, National School of Veterinary Medicine of Sidi Thabet, University of Manouba, Manouba 2010, Tunisia; 5 Department of Basic Sciences, Higher Institute of Biotechnology of Sidi Thabet, University of Manouba, Manouba 2010, Tunisia

**Keywords:** helminth, livestock-wildlife interface, One Health, endangered species conservation, pastoral systems, Uzbekistan

## Abstract

**Objectives:** Helminth infections impose major health burdens on livestock and threaten wildlife at the livestock–wildlife interface in Central Asian drylands. Despite rising concern over parasite transmission between domestic and wild ungulates, integrated surveys remain limited. This study characterized helminth species richness, prevalence, and infection patterns in domestic ruminants (sheep, goats, and cattle) and critically endangered wild ungulates (*Ovis ammon bocharensis, Gazella subgutturosa*, and *Cervus hanglu yarkandensis*) in the Bukhara region of Uzbekistan.

**Materials and Methods:** From spring to summer 2025, complete necropsies (*n* = 51) and organ-specific examinations (*n* = 178) were conducted on domestic ruminants, while coprological surveys (*n* = 256) were performed on wild ungulates. Parasite identification followed standard morphological keys.

**Results:** Cattle had the highest helminth species richness (28), followed by sheep (24) and goats (21). Zoonotic species, including *Echinococcus granulosus, Taenia hydatigena* (metacestodes), and *Fasciola* spp., were detected across hosts. Coprological screening revealed helminths in Goitered Gazelle (32.6%), Bukhara Argali (23.3%), and Bukhara Deer (6.5%). Necropsy and coprological data provide complementary insights but limit direct prevalence comparisons due to differing diagnostic sensitivities.

**Conclusions:** This study provides the first comprehensive helminth baseline for the region, informing targeted anthelmintic strategies, One Health surveillance of zoonotic helminths, conservation management of endangered ungulates, and understanding of parasite dynamics at livestock-wildlife interfaces in arid ecosystems.

## 1. Introduction

Global intensification of livestock production and pastoral land use has reshaped ecological communities, often facilitating the emergence and persistence of parasitic infections in ruminants [1, 2]. Central Asian rangelands, spanning over 250 million hectares from Kazakhstan to Uzbekistan, constitute one of the world’s major pastoral regions, where human-mediated changes in land tenure and livestock management continue to reshape host-parasite dynamics [3]. In Uzbekistan, the post-Soviet transition catalyzed widespread conversion of communal pastures into private holdings, coupled with the importation of high-yield livestock breeds and the expansion of veterinary services [3]. These socioeconomic and management shifts, mirroring broader transformations across Central Asian pastoral systems, underscore the urgent need to understand how human activities influence helminth faunal complexes in both domestic and wild ungulates at the livestock-wildlife interface [4].

The Bukhara, Navoi, and Samarkand regions of Uzbekistan constitute a major ruminant production area where pastoral livelihoods and wildlife conservation intersect. These regions support dense populations of sheep, goats, and cattle while harboring remnant populations of globally significant endangered ungulates: the Bukhara Argali (*Ovis ammon bocharensis*), listed under the Convention on Migratory Species (CMS) and classified as Near Threatened by IUCN due to competition with livestock and habitat loss; the Goitered Gazelle (*Gazella subgutturosa*), an iconic Central Asian dryland species facing population decline; and the Bukhara Deer (*Cervus hanglu bactrianus*), a subspecies of the Central Asian red deer complex with critically fragmented populations across the region. This livestock-wildlife interface creates conditions for cross-species helminth transmission, with implications extending beyond regional borders. As helminth infections can severely reduce livestock productivity and pose zoonotic threats to humans, concerns are particularly acute in Central Asia, where neglected tropical diseases persist. Comprehensive surveillance of parasite communities across this agro-ecological landscape is critical not only for local animal health management but also for understanding parasite dynamics at livestock-wildlife interfaces in arid ecosystems globally [5, 6].

Early investigations of ruminant helminths in Uzbekistan began with the preliminary surveys by Matchanov et al. [7] in sheep and goats, which documented only a limited number of species. Expanded these findings by reporting additional cestode and nematode taxa in small ruminants. More recently, Goyipova [8] provided an updated checklist of cattle helminths in the Zarafshan Valley, yet these works focused exclusively on single host groups and did not integrate wildlife hosts or account for contemporary livestock management changes.

Critically, no previous study has examined helminth communities at the livestock-wildlife interface in this region, despite growing recognition that such interfaces constitute hotspots for parasite spillover and emergence globally. Consequently, a holistic understanding of helminth diversity, prevalence, and transmission dynamics across domestic and wild ruminants in the Bukhara region remains lacking [8], limiting both evidence-based parasite control in pastoral systems and informed conservation management of endangered ungulates.

Addressing this knowledge gap is essential for forecasting the emergence of high-risk helminthiasis foci, devising effective pasture management strategies, and informing One Health approaches to zoonotic parasite surveillance. By integrating necropsy-based surveys of sheep, goats, and cattle with coprological examinations of Argali, Gazelle, and Deer, our study provides the first comprehensive assessment of helminth communities across coexisting domestic and wild ruminants within this Central Asian dryland ecosystem.

The primary objectives of this investigation are articulated in a cohesive framework: first, to quantify helminth species richness and characterize community composition in both livestock and endangered wildlife hosts under contemporary grazing systems, establishing a parasitological baseline for a critically under-studied region; second, to determine the prevalence and intensity of key helminth taxa using standardized parasitological techniques while explicitly accounting for methodological differences between necropsy and coprological approaches; third, to assess the presence and potential spillover risk of zoonotic helminths (including *Echinococcus granulosus, Taenia* spp., and *Fasciola* spp.) across the livestock-wildlife interface; and fourth, to translate these findings into evidence-based recommendations for integrated parasite control and pasture management that support animal productivity, public health, and conservation of globally significant endangered ungulate populations. These objectives position this study within the broader context of understanding parasite ecology at livestock-wildlife interfaces in drylands worldwide, with particular relevance to regions experiencing rapid pastoral system transformation.

## 2. Materials and Methods

### 2.1. Ethical clearance

All animal procedures were performed in accordance with the Ethics Committee’s approval (Directive 2010/63/EU) and international animal welfare guidelines. Written informed consent was obtained from livestock owners before collecting any samples.

### 2.2. Study area and sampling design

**Figure 1.**
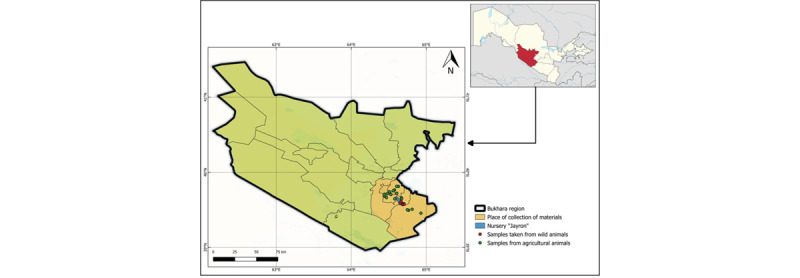
Map of the Bukhara region, Uzbekistan, showing the studied sites.

The Bukhara region of Uzbekistan encompasses semi-arid steppes, riparian zones (rivers and canals), and water reservoirs, providing key forage and habitat for both domestic and endangered wild ruminants. Six districts (Kagan, Jandar, Alat, Karaulbazar, Vabkent, and Bukhara) were selected to capture diverse ecological microenvironments (Figure 1). Between March and August 2025, domestic sheep, goats, and cattle were sampled at ten strategically distributed farms using a stratified design by host species, age (2–5 years), and season. Wild Argali (*O. ammon bocharensis*), Goitered Gazelle (*G. subgutturosa*), and Bukhara Deer (*C. hanglu bactrianus*) housed at the Jeyran conservation nursery in Kagan district were surveyed via systematic coprological collection. Sample sizes were determined by power analysis (Student’s *t*-test, *α* = 0.05, *β* = 0.2), assuming a 30% infection prevalence to achieve ± 5% precision at the 95% confidence level.

### 2.3. Animal selection and ethical considerations

Domestic ruminants were eligible if they were residents of Bukhara for ≥ 10 months, had not been treated with anthelmintics for ≥ 8 weeks, were aged 1–5 months, and were clinically healthy. Exclusion criteria included recent anthelmintic or antibiotic treatment, advanced pregnancy, or incomplete identification. All procedures complied with EU Directive 2010/63/EU and international welfare guidelines, with ethics committee approval and written owner consent obtained prior to sampling.

### 2.4. Parasitological procedures

#### 2.4.1. Domestic ruminants

Complete helminthological necropsies were performed on 51 animals (23 sheep, 15 goats, and 13 cattle) according to the method of Skrjabin [9]. Animals originated from Yamanjar (Boisov Jalol farm) and Kirlishon (Yosh Botir farm) under veterinary oversight. Additionally, targeted organ examinations were conducted on 178 animals (85 sheep, 38 goats, and 55 cattle), inspecting the esophagus, stomach, intestines, trachea, bronchi, lungs, liver, heart, brain, and subcutaneous muscle. Organs were collected 2–4 h postmortem, stored separately at 4°C in labeled containers, and processed under a stereomicroscope to ensure parasite integrity.

#### 2.4.2. Wild ruminants

Noninvasive coprological sampling of 256 fresh fecal deposits (collected within 22–23 h of defecation) was carried out at the conservation nursery. Samples were labeled with species, individual ID (when known), date, and environmental conditions; preserved using the Baermann technique; and refrigerated at 4°C for processing within 24–48 h.

Critical methodological note: Owing to the differing diagnostic sensitivities of necropsy (adult helminth recovery) versus coprological (egg/larval detection) approaches, data from domestic and wild hosts are presented separately and interpreted qualitatively, as direct quantitative comparisons are not valid.

### 2.5. Laboratory methods and quality control

Helminth recovery and processing adhered to Skrjabin’s protocols, with enhancements to ensure consistency and traceability. Adult worms were gently extracted with fine-tipped forceps, rinsed in 0.9% saline at 37°C, relaxed in 0.75% saline for 30–40 sec, then fixed and mounted as permanent slides. Temporary mounts in 70% ethanol facilitated preliminary morphological sorting before detailed taxonomic analysis. Coprological examinations combined direct wet mounts with sedimentation and high-density flotation to recover trematode ova and light eggs, respectively, followed by Baermann-style larval culture for nematode detection.

To minimize observer bias, two independent parasitologists examined each specimen, with discrepant identifications reconciled by consensus. Species were identified using Ivashkin & Mukhammadiev [10], Ivashkin et al. [11], Anderson [12], and Azimov et al. [3], based on morphometric criteria (e.g., body length/width, spicule and gubernaculum dimensions, scolex and proglottid measurements, and sucker size). Microscopes (LOMO C-10 stereoscope, Olympus CK-2TR, and Motic NLCD-307 B) were calibrated against certified stage and eyepiece micrometers. Representative voucher specimens (e.g., *Nematodirus* sp. BU-SNJ-1-PV837616; *N. battus* BU-SNJ-2-PX046409; *N. battus* BU-SNJ-3-PX046408) were deposited in the National Helminth Collection for future verification [13].

### 2.6. Data analysis and statistical treatment

Prevalence (%) and mean intensity (worms per infected host) were computed for each host species, with 95% confidence intervals estimated via the Wilson score method. Given overdispersion and zero inflation in count data, infection intensity was modeled using negative binomial generalized linear models (GLMs) with a log link, including host species as a fixed effect and farm/facility as a random intercept. Pairwise comparisons employed Tukey-adjusted contrasts. Prevalence differences were assessed using logistic regression for binary infection status, incorporating host species and age as covariates, and controlling for multiple testing *via* the Benjamini-Hochberg false discovery rate. Species richness and diversity were quantified using Shannon and Simpson indices, compared among hosts using a permutation-based analysis of variance, and community similarity was evaluated using Jaccard and Sørensen coefficients. All analyses were performed in R v4.2 (packages “MASS,” “vegan,” and “lme4”), with significance set at *p* < 0.05 after adjustment.

## 3. Results

### 3.1. Species richness across host species

**Figure 2.**
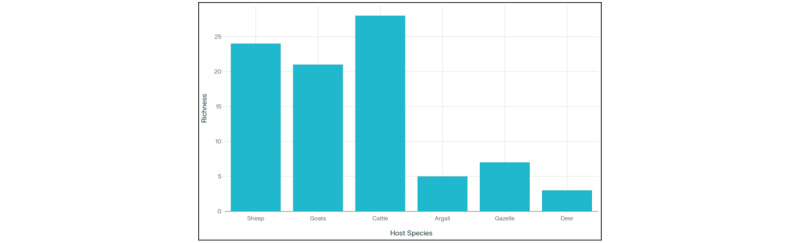
Helminth species richness per host species. This bar chart, rendered on a completely white background, illustrates the number of helminth species identified in each host species: sheep (24 species), goats (21 species), cattle (28 species), argali (5 species), gazelle (7 species), and deer (3 species). The figure highlights the greater parasite diversity in domestic ruminants compared to wild species.

Helminth species richness was substantially higher in managed livestock than in wildlife (Figure 2). Among domestic hosts, cattle (*n* = 13 necropsies) harbored the greatest diversity (28 species), followed by sheep (24 species, *n* = 23) and goats (21 species, *n* = 15). The limited sample size in cattle necropsies reduces statistical power to detect subtle prevalence differences, warranting expanded sampling in future studies. By contrast, wild ruminants exhibited markedly lower richness: Goitered Gazelle supported 7 species, Argali 5, and Bukhara Deer only 3.

### 3.2. Class composition in cattle

The composition of helminth classes in cattle was dominated by nematodes, which accounted for 48% of species (15/28), followed by cestodes (32%, 8/28) and trematodes (20%, 5/28).

### 3.3. Prevalence and intensity of cestodes in domestic ruminants

**Figure 3.**
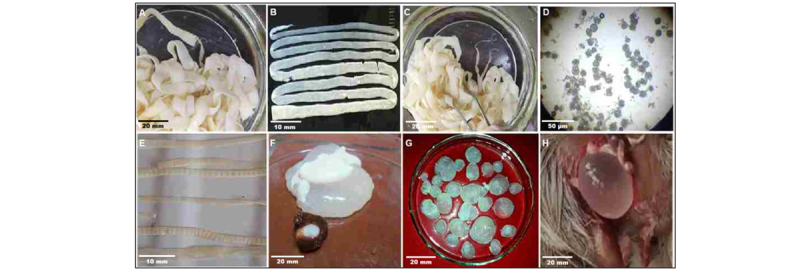
Diversity of cestode helminths recovered from analyzed domestic and wild ruminants. Representative morphology of eight cestode species collected from ruminant hosts. A) *Moniezia expansa*: whole adult tapeworm; B) *Moniezia expansa*: lateral view of adult proglottids; C) *Moniezia benedeni*: strobila; D) *Moniezia benedeni*: eggs; E) *Thysaniezia giardia*: strobila; F) *Taenia hydatigena*: cysticercus (metacestode) in hepatic tissue; G) *Echinococcus granulosus*: hydatid cyst (metacestode); H) *Multiceps multiceps*: larval coenurus (*Coenurus cerebralis*). Scale bars: A, C = 20 mm; B, E = 10 mm; D = 50 µm; F-H = 20 mm.

Cestode infections were detected in all domestic hosts. *Moniezia expansa* had the highest prevalence: 53.8% in cattle (95% CI 29.1–76.8), 26.1% in sheep (12.5–46.5), and 26.7% in goats (10.9–52.0). Logistic regression controlling for host species and farm showed no significant association between host species and *M. expansa* prevalence (aOR cattle vs. sheep = 2.5, 95% CI 0.7–8.9; cattle vs. goats = 2.3, 0.6–9.1; *p* > 0.1). Other cestodes (*M. benedeni, T. giardi*, and larval *E. granulosus*) had prevalence ranges of 17–39% in cattle, 7–20% in sheep, and 7–20% in goats, with no significant host effect (all *p* > 0.1; Table 1). Mean infection intensities per infected host, modeled using negative-binomial GLMs, ranged from 2 to 15 worms depending on species and host, with no significant differences among host species (*p* > 0.05) (Figure 3).

**Table 1.** Prevalence, mean intensity, and host-effect statistics for cestode infections in domestic ruminants.

Cestode species	Host (*n*)	Prevalence % (95% CI)	Mean intensity ± SD (range)	aOR (95% CI) cattle vs. sheep	aOR (95% CI) cattle vs. goats	*p* (FDR)
*Moniezia expansa*	Sheep (23)	26.1 (12.5–46.5)	3.2 ± 4.5 (1–19)	Reference	Reference	–
	Goats (15)	26.7 (10.9–52.0)	3.4 ± 4.1 (1–5)	–	Reference	1.00
	Cattle (13)	53.8 (29.1–76.8)	4.3 ± 6.1 (1–7)	2.5 (0.7–8.9)	2.3 (0.6–9.1)	0.18
*Moniezia benedeni*	Sheep (23)	17.4 (6.8–37.1)	5.6 ± 3.9 (3–15)	Reference	Reference	–
	Goats (15)	20.0 (7.0–43.7)	2.3 ± 1.0 (1–3)	–	Reference	0.89
	Cattle (13)	38.5 (17.7–64.5)	4.8 ± 2.9 (1–8)	3.2 (0.8–12.4)	2.0 (0.5–8.1)	0.21
*Thysaniezia giardi*	Sheep (23)	8.7 (2.4–24.6)	7.6 ± 3.2 (1–13)	Reference	Reference	–
	Goats (15)	6.7 (0.8–30.5)	3.5 ± 0.5 (3–4)	–	Reference	1.00
	Cattle (13)	15.4 (4.3–42.2)	4.2 ± 1.2 (3–5)	1.9 (0.4–8.5)	2.3 (0.5–10.2)	0.65
*Echinococcus granulosus*ᵃ	Sheep (23)	21.7 (9.8–41.9)	10.2 ± 6.8 (1–20)	Reference	Reference	–
	Goats (15)	20.0 (7.0–43.7)	1.5 ± 0.5 (1–2)	–	Reference	1.00
	Cattle (13)	30.8 (12.7–57.6)	9.1 ± 4.3 (5–13)	1.6 (0.5–5.3)	1.8 (0.5–6.4)	0.67

^a^, larval stage in intermediate host tissues. aOR: adjusted odds ratio from logistic regression (reference: sheep for cattle vs. sheep; goats for cattle vs. goats), with farm as a random effect. *p* (FDR): *p*-value after Benjamini-Hochberg correction.

### 3.4. Prevalence and intensity of trematodes in domestic ruminants

Trematode infections were most frequent in cattle with *F. gigantica* (46.2%, 23.2–70.9) and *F. hepatica* (30.8%, 12.7–57.6), compared with sheep (*F. gigantica* 26.1%, 12.5–46.5; *F. hepatica* 21.7%, 9.8–41.9) and goats (*F. gigantica* 26.7%, 10.9–52.0; *F. hepatica* 20.0%, 7.0–43.7).

**Figure 4.**
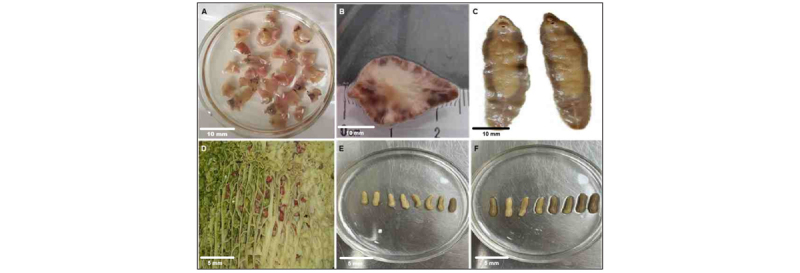
Morphological variation among trematode flukes recovered from domestic and wild ruminants. Representative morphology of six trematode species collected from ruminant hosts: (A) *Fasciola hepatica*, adult flukes; (B) *F. hepatica*, dorsal view; (C) *Fasciola gigantica*, ventral view; (D) *Calicophoron calicophorum*, in situ within host organ; (E) *C. calicophorum*, adult flukes; (F) *Gastrothylax crumenifer*, adult flukes. Scale bars: A–C = 10 mm; D–F = 5 mm.

Logistic models adjusted for farm effects indicated no significant host-species differences (aORs 1.6–2.4; all *p* (FDR) > 0.1). Mean infection intensities per host, analyzed with negative-binomial GLMs including seasonal covariates, ranged from 1 to 12 flukes depending on species and host, without significant differences among hosts (*p* (FDR) > 0.05) (Table 2, Figure 4).

**Table 2.** Prevalence, mean intensity, and host-effect statistics for major nematode infections in domestic ruminants.

Nematode species	Host (*n*)	Prevalence % (95% CI)	Mean intensity ± SD (range)	aOR (95% CI) cattle vs. sheep	aOR (95% CI) cattle vs. goats	*p* (FDR)
*Trichocephalus ovis*	Sheep (23)	47.8 (29.2–67.0)	12.3 ± 18.5 (1–89)	Reference	Reference	–
	Goats (15)	66.7 (43.8–84.4)	3.8 ± 1.2 (1–5)	–	Reference	0.42
	Cattle (13)	69.2 (42.4–87.3)	45.9 ± 40.7 (3–112)	2.1 (0.6–7.4)	1.3 (0.4–4.3)	0.37
*Strongyloides papillosus*	Sheep (23)	47.8 (29.2–67.0)	54.3 ± 45.6 (6–152)	Reference	Reference	–
	Goats (15)	53.3 (30.1–75.2)	46.7 ± 43.2 (5–140)	–	Reference	1.00
	Cattle (13)	69.2 (42.4–87.3)	112.3 ± 123.4 (3–390)	2.3 (0.7–7.5)	1.5 (0.5–4.6)	0.37
*Dictyocaulus filaria*	Sheep (23)	52.2 (32.9–70.8)	12.0 ± 10.3 (2–29)	Reference	Reference	–
	Goats (15)	40.0 (19.8–64.3)	4.7 ± 3.2 (1–11)	–	Reference	0.58
	Cattle (13)	53.8 (29.1–76.8)	17.2 ± 9.5 (3–35)	1.0 (0.3–3.5)	1.4 (0.4–4.8)	0.95
*Marshallagia marshalli*	Sheep (23)	56.5 (36.8–74.4)	123.5 ± 140.2 (5–550)	Reference	Reference	–
	Goats (15)	60.0 (35.7–80.2)	45.8 ± 42.3 (3–150)	–	Reference	0.88
	Cattle (13)	69.2 (42.4–87.3)	168.4 ± 189.1 (1–550)	1.7 (0.6–5.1)	1.2 (0.4–3.7)	0.57

aOR from logistic regression with farm as random effect (reference = sheep for cattle vs. sheep; goats for cattle vs. goats), *p* (FDR) by Benjamini-Hochberg correction.

### 3.5. Prevalence and intensity of nematodes in domestic ruminants

**Figure 5.**
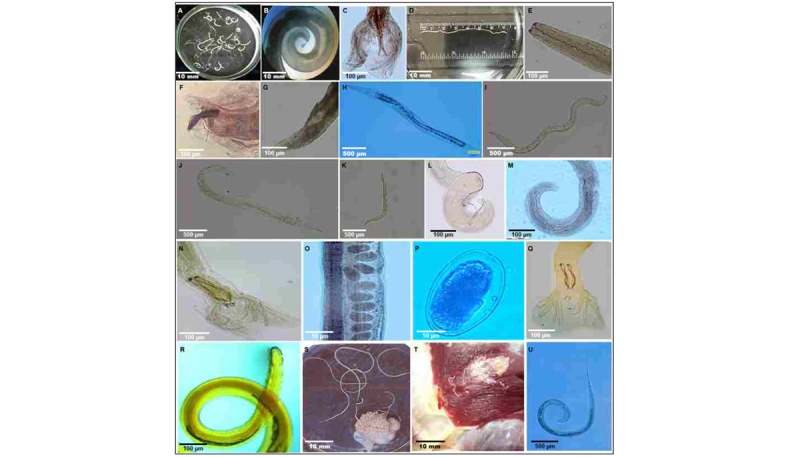
Adult and larval nematode forms recovered from domestic and wild ruminants. Representative morphology of eighteen nematode taxa at different life stages: (A) *Trichocephalus ovis*, adult; (B) *Trichocephalus skrjabini*, adult; (C) *Haemonchus contortus*, posterior end of male; (D) *Dictyocaulus viviparus*, adult; (E) *Dictyocaulus filaria*, anterior end; (F) *D. filaria*, posterior end of male; (G) *D. filaria*, posterior end of female; (H) *Strongyloides papillosus*, free-living female; (I) *Strongyloides stercoralis*, free-living female; (J) *S. stercoralis*, free-living male; (K) *S. stercoralis*, larva; (L) *Parabronema skrjabini*, posterior end of male; (M) *P. skrjabini*, posterior end of female; (N) *Marshallagia marshalli*, posterior end of male; (O) *M. marshalli*, female; (P) *M. marshalli*, egg; (Q) *Marshallagia mongolica*, posterior end of male; (R) *Gongylonema pulchrum*, anterior end; (S) *Setaria labiato-papillosa*, general view; (T) *Onchocerca gutturosa*, in host tissue; (U) *Nematodirus battus*, larva. Scale bars: A, B, D, S, T = 10 mm; H-K, U = 500 µm; C, E-G, L-N, Q, R = 100 µm; O-P = 50 µm.

Nematode infections were the most prevalent helminth group in domestic ruminants. *T. ovis* prevalence was 47.8% in sheep, 66.7% in goats, and 69.2% in cattle; logistic models adjusting for farm effects showed no significant host-species differences (aOR cattle vs. sheep = 2.1, 95% CI 0.6–7.4; cattle vs. goats = 1.3, 0.4–4.3; *p* (FDR) > 0.1). *Strongyloides papillosus* followed similar patterns (47.8%, 53.3%, 69.2%; aORs 1.5–2.3, *p* (FDR) > 0.1). Mean infection intensities, modeled using negative-binomial GLMs, ranged widely (e.g., *T. ovis* 12.3 ± 18.5 in sheep vs. 45.9 ± 40.7 in cattle) but did not differ significantly among hosts (*p* (FDR) > 0.05) (Table 3, Figure 5).

### 3.6. Coprological findings in wild ruminants

Coprological surveys showed lower infection rates in wild hosts compared with domestic ruminants. Bukhara Argali had 23.3% prevalence (*n* = 60; 95% CI 14.3–35.6), Goitered Gazelle 32.6% (*n* = 89; 23.5–43.1), and Bukhara Deer 6.5% (*n* = 107; 3.1–13.1). Larval *S. stercoralis* was detected in Gazelle (11.2%; 6.0–20.0) and Deer (2.8%; 0.8–8.7), while *S. papillosus* occurred only in Argali (5.0%; 1.6–13.5). Egg counts of *F. hepatica* ranged from 8.3% to 16.7% across all three species, and *T. ovis* eggs were recorded only in Gazelle (4.5%) and Argali (1.7%), reflecting distinct host-parasite associations in wild populations.

## 4. Discussion

Our survey builds upon over 150 years of helminthological research in Central Asia, beginning with A.P. Fedchenko’s mid-19th-century collections from wild and domestic ruminants in the Bukhara region [14]. Early taxonomic descriptions were provided by Krabbe [15] and Linstow [16, 17], while Skrjabin [18] established standardized morphological keys and catalogued 16 helminth species in sheep, with emphasis on trichostrongylid nematodes. Later expeditions by Petrov & Shekhovtseva [19] and Ershov [20] expanded the fauna to 26 species across sheep and cattle. Sultanov et al. [21] synthesized decades of research, documenting 34 helminth species in domestic ruminants. Wildlife hosts remained understudied until Dadaev [22] and Turemuratov [23] documented approximately 20 helminth species in Cervidae and Bovidae. Our study addresses this gap by concurrently assessing helminth communities in sheep, goats, cattle, and three endangered ungulates, Bukhara Argali (*O. ammon bocharensis*), Goitered Gazelle (*G. subgutturosa*), and Bukhara Deer (*C. hangl u bactrianus*), under contemporary ecological and veterinary management. This approach provides the first comprehensive parasitological profile at the livestock-wildlife interface in Central Asian drylands.

Consistent with historical patterns [18, 21], domestic ruminants displayed higher helminth species richness (sheep 24, goats 21, cattle 28) than wild hosts (Argali 5, Gazelle 7, Deer 3; Figure 2). This disparity likely reflects ecological differences: high livestock densities and communal grazing increase transmission opportunities; shared pastures facilitate cross-species parasite exchange; repeated anthelmintic treatments may select for resistant lineages [24, 25]; and water and feeding hotspots concentrate infective stages, ensuring year-round transmission.

The predominance of nematodes in cattle (48% of recorded species; Figure 2) echoes Skrjabin’s [18] early observations of trichostrongylid dominance and aligns with Sultanov et al.’s [21] comprehensive documentation of the diverse nematode complex in Central Asian Bovidae. This pattern persists globally, as cattle grazing systems typically favor nematode transmission through their requirement for moist microhabitats where free-living larval stages develop [26, 13, 3]. In contrast, the limited helminth diversity in wild populations reflects lower host densities, spatially dispersed grazing patterns that reduce pasture contamination, and restricted interspecific transmission due to habitat separation between Argali (montane grasslands), Gazelle (riparian zones), and Deer (forest-steppe mosaics).

A critical limitation acknowledged in our study is the fundamental incomparability of necropsy-derived data from domestic hosts and coprological results from wildlife. Multiple validation studies have demonstrated that coprological examinations systematically underestimate true helminth prevalence by 50–90% due to intermittent egg shedding, low sensitivity for pre-patent infections, and inability to detect sterile or immature worms. This methodological constraint prevents direct quantitative comparisons of species richness or prevalence between domestic and wild hosts, as observed differences may reflect diagnostic sensitivity rather than genuine ecological patterns. Consequently, our higher richness estimates in livestock (24–28 species) versus wildlife (3–7 species) must be interpreted cautiously, recognizing that a complete necropsy would likely reveal additional helminth species in wild populations currently undetected by fecal examination.

Despite this limitation, coprological surveys remain the only ethically and logistically feasible approach for monitoring endangered species under conservation management, where sacrificial sampling is prohibited. Future research should prioritize molecular coprodiagnostic methods (e.g., metabarcoding and species-specific qPCR) that offer greater sensitivity than traditional microscopy and enable the detection of cryptic species complexes within morphologically similar taxa, such as *Strongyloides* and *Trichocephalus*. Such molecular approaches would refine our understanding of helminth diversity at the livestock-wildlife interface while adhering to noninvasive sampling protocols required for threatened species.

Contrary to expectations based on host phylogenetic distance, prevalence and infection intensity analyses revealed no significant host-species effects for major cestode, trematode, and nematode taxa after adjusting for farm or nursery facility and correcting for multiple testing via false discovery rate (Tables 1–3). Shared grazing areas and overlapping anthelmintic protocols likely homogenize infection risks for *Moniezia* spp., *Fasciola* spp., *T. ovis*, and *S. papillosus* across sheep, goats, and cattle [27, 28]. These results substantiate Ershov’s [20] early reports of similar parasite burdens in co-grazing species and underscore the epidemiological reality that in intensive pastoral systems, host species identity becomes secondary to management factors (stocking density, pasture rotation schedules, and anthelmintic treatment frequency) in determining infection patterns.

The detection of zoonotic species, including larval *E. granulosus* (20–31% prevalence across hosts), *T. hydatigena* metacestodes, and *Fasciola* spp. (21–46% prevalence), highlights the public health dimension of livestock helminth surveillance in Central Asia, a region where neglected tropical diseases persist, and rural communities maintain close contact with ruminants. The comparable prevalence of these zoonotic taxa across domestic species suggests that targeted control strategies must address all three host groups simultaneously rather than focusing on single-species interventions, reinforcing the One Health imperative for integrated human-animal-environmental health surveillance.

Coprological surveys confirmed significantly lower helminth detection rates in wild populations (Argali 23.3%, Gazelle 32.6%, Deer 6.5%) compared to domestic necropsy data, with Goitered Gazelle exhibiting the highest wild host prevalence (*p* = 0.011 versus Argali) and Bukhara Deer the lowest (*p* < 0.001 versus both Argali and Gazelle). Beyond methodological differences in sensitivity, these patterns likely reflect genuine ecological variation in exposure risk driven by habitat use and foraging behavior. Goitered Gazelle’s preference for riparian grazing near water sources and irrigation canals places them in contact with moist microhabitats where infective nematode and trematode larvae concentrate, whereas Bukhara Deer’s forest-steppe habitat provides drier conditions less conducive to parasite transmission [29, 30].

The detection of *Strongyloides* spp. larvae in Gazelle (11.2%) and Deer (2.8%), alongside *F. hepatica* eggs across all three wild species (8.3–16.7%), warrants molecular confirmation to resolve cryptic host-parasite associations and potential spillover from sympatric livestock populations. Dadaev’s [22] earlier records of similar taxa in wild Bovidae and Cervidae suggest long-standing endemic circulation, yet the contemporary expansion of livestock into former wildlife habitats raises concerns about anthropogenic amplification of parasite transmission at newly formed interfaces. From a conservation perspective, targeted surveillance in Goitered gazelle populations, already classified as vulnerable by IUCN, may preempt parasite-mediated population declines analogous to those documented in other threatened ungulates where helminth infections synergize with habitat loss and nutritional stress [31].

High livestock helminth burdens (Tables 1–3) necessitate region-adapted interventions for Bukhara’s steppe pastures and 40°C summers. Pasture rotation every 4–6 weeks avoids the Strongyloides transmission peak (June–August), while 500 m exclusion zones around the Jeyran nursery minimize livestock-wildlife spillover. FAMACHA^©^ scoring identifies anemic Argali/Gazelle for targeted treatment, and annual sorghum cropping in irrigation canals disrupts *Fasciola* cycles. Targeted selective treatment (TST) of the top 20% egg shedders preserves refugia, reducing anthelmintic use 30–50% while controlling parasite loads [21]. Veterinary-conservation collaboration is essential.

**Table 3.** Comparative prevalence, species richness, and host-effect statistics for overall helminth infections.

Host species	*n*	Prevalence % (95% CI)	Species richness	aOR (95% CI) *vs*. Sheep	aOR (95% CI) vs. Goats	aOR (95% CI) vs. Argali	*p* (FDR)
Sheep (*O. aries*)	23	100.0 (85.2–100.0)	24	Reference	–	–	–
Goats (*C. hircus*)	15	86.7 (62.1–96.3)	21	0.13 (0.01–1.19)	Reference	–	0.06
Cattle (*B. taurus*)	13	100.0 (75.3–100.0)	28	–	–	–	–
Bukhara Argali	60	23.3 (14.3–35.6)	5	–	–	Reference	–
Goitered Gazelle	89	32.6 (23.5–43.1)	7	–	–	1.56 (0.67–3.62)	0.26
Bukhara Deer	107	6.5 (3.1–13.1)	3	–	–	0.23 (0.09–0.58)	< 0.001

aOR from logistic regression with farm or nursery as random effect (domestic: reference = sheep; wild: reference = Argali); *p* (FDR) by Benjamini-Hochberg.

Targeted selective treatment (TST), whereby only animals exceeding predefined fecal egg count thresholds or exhibiting clinical signs receive anthelmintics, preserves a refugia population of susceptible parasites that dilutes resistant genotypes emerging in treated hosts.

Implementation of TST requires regular fecal egg count monitoring and producer training, but field trials in Mediterranean and tropical systems have demonstrated 30–50% reductions in anthelmintic use without compromising production metrics. Given the accelerating reports of anthelmintic resistance in Central Asian livestock populations, proactive adoption of TST combined with pasture management may forestall the treatment failures increasingly reported in intensive systems globally.

For wildlife management, minimizing spatial and temporal overlap between livestock grazing and key wildlife foraging areas could decrease bidirectional spillover of generalist helminths that infect both domestic and wild hosts. Establishment of grazing exclusion zones around conservation facilities like the Jeyran nursery, coupled with seasonal livestock movement schedules that avoid wildlife breeding and birthing periods when nutritional stress elevates infection susceptibility, would reduce interface-mediated transmission. Complementary measures, such as anthelmintic treatment of livestock herds prior to entering interface zones, can lower pasture contamination, though this must be balanced against selection pressures for resistance.

Ultimately, effective management at livestock-wildlife interfaces demands cross-sector collaboration among veterinary services, conservation authorities, and pastoral communities, embodying the participatory governance principles central to One Health approaches.

While our study provides new insights into helminth communities in Bukhara ruminants, several limitations should be noted. Sampling was opportunistic, constrained by the farm owner’s consent and access to the wildlife facility, which may bias prevalence estimates and host representation. Morphological identification alone, without molecular confirmation, may have missed cryptic species complexes, particularly within *Strongyloides, Trichocephalus*, and *Nematodirus*. In addition, incomplete metadata on host age, sex, and nutritional status prevented assessment of demographic risk factors. Future studies should implement stratified random sampling with adequate statistical power, incorporate molecular tools such as DNA barcoding or metabarcoding to resolve cryptic diversity, and collect detailed host metadata. Longitudinal monitoring across seasons could clarify the temporal dynamics of helminth transmission, and experimental studies on grazing management or livestock-wildlife interfaces would help establish causal links to infection patterns.

## 5. Conclusions

This study provides the first integrated survey of helminth communities in domestic and wild ruminants of the Bukhara region, establishing a regional baseline for future research. Domestic livestock, sheep, goats, and cattle exhibited higher species richness and prevalence than wild ruminants, with cattle showing the greatest diversity (28 species), largely dominated by nematodes (48%). Infection prevalence and intensity were broadly similar among domestic hosts, whereas wild ruminants had lower overall prevalence, with the Goitered gazelle showing the highest infection rate. These findings highlight the influence of livestock management on helminth transmission and the need for integrated control measures, including rotational grazing, targeted anthelmintic treatment, and pasture management. They also inform conservation strategies by identifying wildlife species at higher risk and potential parasite spillover from livestock. While morphological identification provided important taxonomic insights, molecular confirmation would further strengthen species resolution, particularly for morphologically similar genera. This work fills key knowledge gaps in Central Asian helminth fauna, updates historical baselines, and provides a foundation for future studies that incorporate molecular diagnostics and multivariate analyses to better understand infection dynamics and guide sustainable interventions.

## Data Availability

The data presented in this study are available from the corresponding author upon reasonable request.

## List of abbreviations

CMS, Convention on Migratory Species; GLMs, generalized linear models; aOR, adjusted odds ratio; FDR, false discovery rate; TST, targeted selective treatment; h, hours; sec, seconds.

## References

[1] Pereira MA, Vila-Viçosa MJ, Coelho C, Santos C, Esteves F, Cruz R (2024). Pulmonary and gastrointestinal parasitic infections in small ruminant autochthonous breeds from the Centre Region of Portugal—A cross-sectional study. Animals.

[2] Rufino-Moya PJ, Leva RZ, Reis LG, García IA, Di Genova DR, Gómez AS (2024). Prevalence of gastrointestinal parasites in small ruminant farms in southern Spain. Animals.

[3] Azimov DA, Dadaev SD, Akramova FD (2015).

[4] Carrera-Játiva PD, Acosta-Jamett G (2023). Influence of habitat alteration on the structure of helminth communities in small mammals: A systematic review and critical appraisal of theory and current evidence. Parasitol Res.

[5] Charlier J, Rinaldi L, Musella V, Ploeger HW, Chartier C, Vineer HR (2020). Initial assessment of the economic burden of major parasitic helminth infections to the ruminant livestock industry in Europe. Prev Vet Med.

[6] Hamid L, Alsayari A, Tak H, Mir SA, Almoyad MAA, Wahab S (2023). An insight into the global problem of gastrointestinal helminth infections amongst livestock: Does nanotechnology provide an alternative?. Agriculture.

[7] Matchanov NM, Dadaev SD, Azimov DA (1986). Helminths of animals of the Northeast of Uzbekistan. Mekhnat, Tashkent, Uzbekistan,.

[8] Goyipova ME (2019). Helminths of large horned animals of the Zarafshan Valley: Fauna, distribution and ecology.

[9] Skrjabin KI (1928).

[10] Ivashkin VM, Mukhammadiev SA

[11] Ivashkin VM, Oripov AO, Sonin MD

[12] Anderson RC

[13] Akramova F, Shakarbaev U, Mirzaeva A, Saidova S, Uralova F, Amirov O (2025). Nematodes fauna of the genus Nematodirus (Nematoda) in domestic and semi-free-ranging ruminants of Central Uzbekistan. Biosyst Divers.

[14] Fedchenko AP (1876).

[15] Krabbe H (1879). Tapeworms (Cestodes). Fedchenko AP. Journey to Turkestan. Zoogeographical Studies. Society of Lovers of Natural Science, Anthropology, and Ethnography, Moscow, Russia.

[16] Linstow O (1883). Nematodes, trematodes and acanthocephalans collected by Prof. Fedtschenko in Turkestan. Arch Naturgesch.

[17] Linstow O (1886). Roundworms and flukes based on materials by A.P. Fedchenko. Zoogeographical Studies. Society of Friends of Natural Science, Anthropology, and Ethnography, Moscow, Russia,.

[18] Skrjabin KI (1916).

[19] Petrov AM, Shekhovtseva ES (1926). On the fauna of parasitic worms of sheep in Turkestan. Proc State Inst Exp Vet Med.

[20] Ershov VS (1933). Work of the 83rd Union Helminthological Expedition in the Kassan Karakul-breeding State Farm of Uzbekistan. Proc Cent Asian Res Inst Vet Med.

[21] Sultanov MA, Azimov DA, Gekhtin VI, Muminov PA (1975).

[22] Dadaev S (1997). Institute of Zoology.

[23] Turemuratov MS (2022).

[24] Winter J, Rehbein S, Joachim A (2018). Transmission of helminths between species of ruminants in Austria appears more likely to occur than generally assumed. Front Vet Sci.

[25] Wu J, Buckley HL, Curry L, Stevenson BA, Schipper LA, Lear G (2021). Livestock exclusion reduces the spillover effects of pastoral agriculture on soil bacterial communities in adjacent forest fragments. Environ Microbiol.

[26] Akramova F, Shakarbaev U, Mirzayeva A, Saidova S, Akbarova M, Uralova F (2025). Helminths of domestic and wild artiodactyls (Mammalia, Artiodactyla) in Uzbekistan. Biosyst Divers.

[27] Abbas I, Hildreth MB (2022). Trichostrongyle infections in domestic ruminants from Egypt: A systematic review and meta-analysis. Vet Parasitol Reg Stud Reports.

[28] Sharma A, Sharma S, Kour S, Avatsingh AU, Perveen K, Alsulami JA (2023). Gastrointestinal nematodes and protozoa in small and large ruminants from rural agro-climatic regions of northern India. Diversity.

[29] Karaer MC, Karataş B, Madak E, Sönmez Hİ, Keskin E, Sarımehmetoğlu HO (2025). Characterizing the helminth community of the mountain gazelle (*Gazella gazella* Pallas, 1766) through DNA metabarcoding. Acta Parasit.

[30] Wang Y, Yuan P, Liu C, Yang Y, Yang W, Zhang D (2025). How the Goitered gazelle (*Gazella subgutturosa*) adapts to isolated island: From the perspective of habitat and food. Wildl Res.

[31] Karaer MC, Sönmez Hİ, Madak E, Kankılıç T, Tavşanoğlu Ç, Sarımehmetoğlu HO (2024). Helminths of captive and free-ranging populations of the mountain gazelle (*Gazella gazella*): Evidence from faecal examination. Vet Med Sci.

